# Lipoprotein transport system Lol may be a selective target for Gram-negative bacteria

**DOI:** 10.3389/fcimb.2024.1463316

**Published:** 2024-10-01

**Authors:** Qiang Yang, Ying Cai, Zhibo Wang, Sifan Guo, Shi Qiu, Aihua Zhang

**Affiliations:** ^1^ GAP Research Center and Graduate School, Heilongjiang University of Chinese Medicine, Harbin, China; ^2^ International Advanced Functional Omics Platform, Scientific Experiment Center, Hainan Engineering Research Center for Biological Sample Resources of Major Diseases, Key Laboratory of Tropical Cardiovascular Diseases Research of Hainan Province, Hainan Medical University, Haikou, China

**Keywords:** Gram-negative bacteria, lipoprotein, gut microbial, antibiotics, resistance

## 1 Introduction

**Figure 1:**
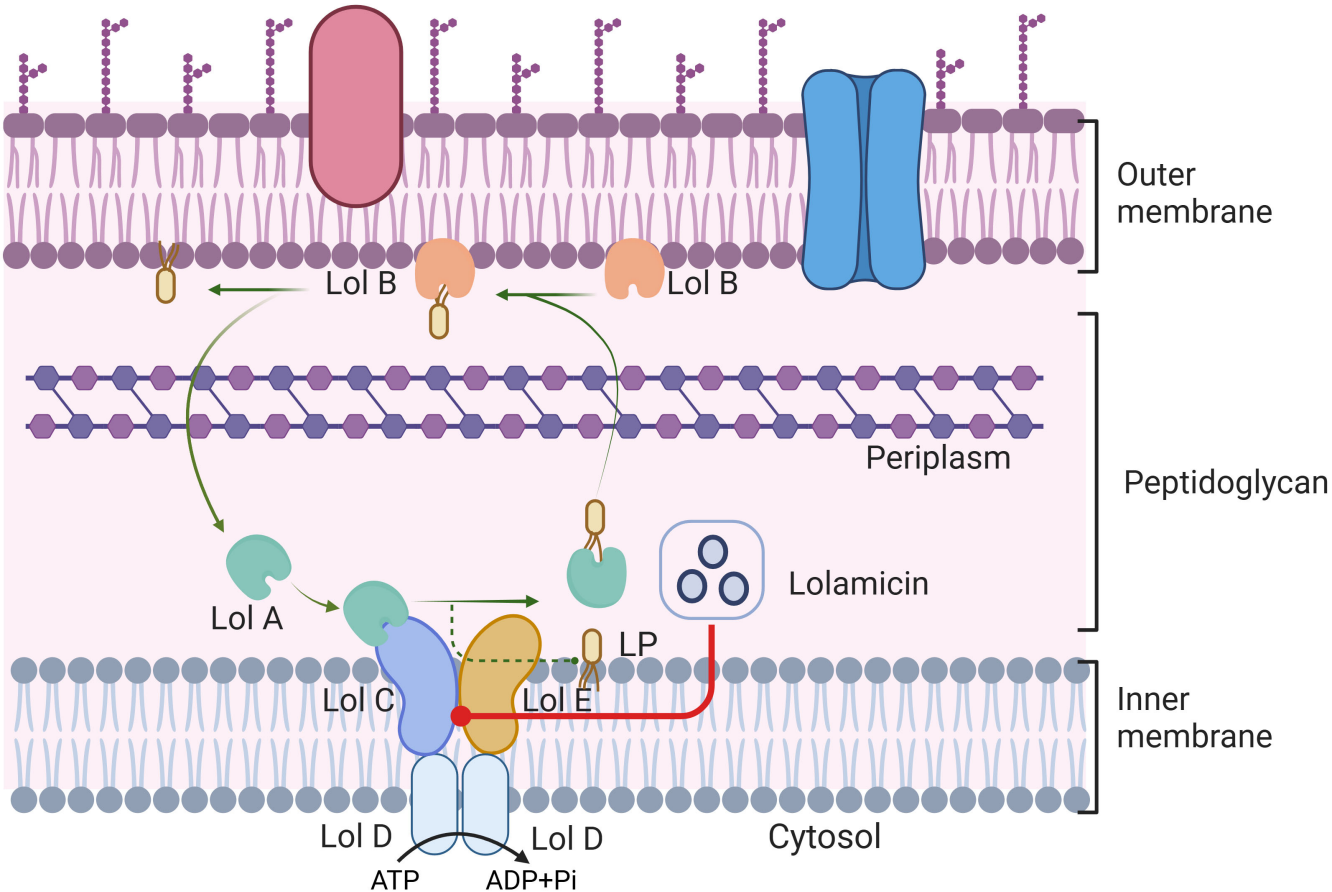
LolABCDE protein mediated lipoprotein transport in Gram-negative bacteria. ATP is hydrolyzed into ADP and inorganic phosphate, lipoprotein is extracted from the cell membrane by LolCDE complex, and transported to the outer membrane protein LolB by LolA. Transport of lipoproteins affected by Lolamicin competitively binding to the LolCDE protein complex in Gram-negative bacteria. This figure is created by BioRender.

In a recent study published in Nature, Munoz et al. have developed the novel antibiotic, lolamicin, which targets the Gram-negative bacterial outer membrane lipoprotein transporter LolCDE. (**Figure 1**) This compound demonstrated efficacy in multiple murine models of acute pneumonia and sepsis, effectively preventing Clostridium difficile infection, as well as maintaining gut microbial stability (Munoz et al., 2024).

In recent years, the World Health Organization has been conducting research on antibiotics and drug-resistant bacteria. Multidrug resistance in Gram-negative bacterial infections poses a significant threat with a high risk of morbidity and mortality. Traditionally, Gram-negative infections (especially their multidrug-resistant strains) have been difficult to treat, mainly due to the significant disadvantages of existing treatment options, such as aminoglycosides and glycocycline, including non-negligible toxicity and possible suboptimal pharmacokinetics at certain infection sites. Consequently, pharmaceutical researchers are eager to discover alternative antibacterial drugs and protocols. While a variety of antibiotics are approved for Gram-negative infection treatment, maintaining the gut microbiome homeostasis after administration remains a challenge. The unique permeability outer membrane structure of Gram-negative bacteria is pivotal in their antibiotic resistance mechanisms. These may involve enzyme inactivation, efflux pumps, porin protein mutations, and target site modifications. Effective targets can be identified through pathways such as efflux pump inhibition, membrane protein modification, lipopolysaccharide synthesis, etc., including recently discovered macrocyclic peptides antibiotics to target lipopolysaccharide synthesis (Luther et al., 2019; Huseby et al., 2024).

Presently, as antibiotic resistance continues to rise, the arsenal of effective antibiotics for Gram-negative bacteria is depleting. It is an urgent need for new antibiotics. Researchers including Munoz suggested that the development of a specific antibiotic without affecting the microbiome should identify its unique target, discriminating it from beneficial commensals (Munoz et al., 2024). Bacterial lipoproteins, a vital component of bacterial cell membranes, is directly involved in the pathogenic mechanisms of numerous infectious agents and serves as an immunostimulatory factor facilitating inflammatory responses, where the lipid transport process of most lipoproteins necessitates the assistance of Lol transport system. It was noted that Lol lipoprotein transport system was essential for bacterial survival in various environments, associated with physiological and pathological mechanisms of Gram-negative bacteria, such as nutrition, stress response, virulence (Tang et al., 2021). The correct localization of lipoprotein in cells is the basis for their physiological function, which depends on the normal operation of the lipoprotein transport system. Lipoprotein synthesis implements the physiological process of bacteria, which makes the lipoprotein transport system necessary for bacterial survival, but there are still problems such as unclear mechanisms of lipoprotein secretion and surface display. Concurrently, during the transportation of lipoproteins, the signal secreted by the N-terminus of mature outer membrane lipoproteins is initially recognized by the LolCDE complex, which participates in transmembrane protein transfer. This transport pathway influences bacterial membrane stress response and further impacts outer membrane synthesis and cell survival (El et al., 2021). Overuse of antibiotics leads to drug and even multi-drug resistance, especially non-selective broad-spectrum antibiotics, which destroy the homeostasis of symbiotic bacteria in the host while killing the bacteria and exacerbating secondary infections. Based on the structure of two LolCDE inhibitors, pyridinepyrazole (Breidenstein et al., 2024) and pyridineimidazole (McLeod et al., 2015; Nayar et al., 2015), the researchers designed a novel LolCDE inhibitor-lolamicin. In drug susceptibility studies of clinical strains, low concentrations of lolamicin were found to be active against clinical strains, highlighting its potential against multiple clinical isolates. The development of this promising new targeted antibiotic could effectively reduce the use of some broad-spectrum antibiotics, improve treatment efficiency, and reduce the risk of drug resistance. However, the mechanism remains obscure.

Potential targets of lolamicin, as determined through methods such as amino acid sequencing, may include lipoprotien transport proteins like LolC and LolD. Observation by focused microscopy revealed that lolamicin therapy could induce a cell swelling phenotype induced by LspA inhibition with Globomycin. However, this phenotype is distinct from that induced by β-lactams, and similar effects are observed in corresponding LolE mutant strains, indirectly confirming that lolamicin can exert its bactericidal effect through targeting the Lol lipoprotein transporter. To further determine the binding check point of lolamicin, the researchers had explored the antibacterial mode of lolamicin on LolCDE using resistant mutants and molecular modeling technology. Four postulated binding modes – BS1/BS2 and two transient sites TS1/TS2 confirmed that lolamicin competitively inhibits lipoproteins via lipopeptide binding sites, disrupting their normal transport. In terms of its mechanism of action, Lolamicin is able to control the transport of LolA lipoprotein to the outer membrane through competitive binding to LolCDE complex protein, thereby disrupting the normal physiological function of Gram-negative bacteria. This competitive binding pattern may be related to the hydrophobic interaction of nonpolar or aromatic groups, providing a plausible explanation for the reduced efficacy caused by the primary amine group in the compound acting on the binding pocket. Subsequently, the researchers further evaluated lolamicin’s *in vivo* efficacy in sepsis and acute pneumonia infection models. They found that lolamicin outperformed pyridinepyrazole in efficacy and demonstrated superiority in various negative infections. High oral bioavailability and robust tolerance are the basis for lolamicin’s *in vivo* efficacy, effectively reducing bacterial load in infected mice and improving mouse survival rates.

Remarkably, regulation and cooperation between gut bacteria are essential for their homeostasis. Long-term use of broad-spectrum antibiotics can cause an imbalance in the gut microbiota, disrupted physiological composition, and pathological combination, leading to clinical symptoms. Munoz et al. studied the effects of various antibiotics on the intestinal microbial community to determine the selectivity of lolamicin against Gram-positive and non-pathogenic Gram-negative bacteria. Significant changes were observed in bacterial communities following broad-spectrum antibiotic amoxicillin and gram-positive targeted antibiotic clindamycin, causing alterations in community diversity and abundance. Conversely, lolamicin therapy maintained relatively stable species abundance and diversity. It showed a significant reduction in bacterial burden and improved survival after treatment, confirming lolamicin’s selective killing effect on pathogenic Gram-negative bacteria. This highlights the potency of lolamicin against Gram-negative bacteria and the significance of microbial community homeostasis post-antibiotic treatment. When antibiotics are used to kill or inhibit bacterial growth, at the same time, bacteria are subjected to screening pressure for antibiotics, and drug-resistant genes are widely spread in various microbial ecological environments, including the human intestinal flora system, causing flora disorders. Notably, the divergence in target sequence homology between pathogens and gut microbes may be a pivotal point for novel selective antibiotic development.

To conclude, it demonstrated lolamicin’s potential as a Gram-negative-specific antibiotic, presenting significant benefits in preserving gut microbiota. Lolamicin selectively eliminates Gram-negative pathogenic bacteria by targeting lipid transport systems, while sparing commensal bacteria, bypassing intestinal dysbiosis and secondary infection problems associated with traditional broad-spectrum antibiotics. However, targeted pharmacokinetic and clinical studies are critical for the clinical application of new drugs, and future research should focus on enhancing lolamicin’s chemical structure, resistance, and potential against other Gram-negative pathogens. Although the research on lolamicin is still in the animal experimental research stage, its clinical potential cannot be ignored, which may provide reference for the development of new drug-resistant antibiotics. Although the transition from preclinical to clinical trials is still unpredictable, the lack of consistency between species remains the biggest obstacle, rather than simple gene regulation and physiological differences. It is undeniable that Munoz et al.’s discovery of a novel Lol transport system inhibitor from preclinical trials still shines a light on more antibiotic researchers. In addition, targeting bacterial lipid transport systems is a novel strategy for the development of novel antimicrobials, and maintaining an optimal balance in the gut microbiota post antibiotic administration warrants equal attention, suggesting that the development of novel targeted antimicrobial drugs could serve as a novel approach to addressing multi-resistant mechanisms of antibiotics.
